# Computer-Assisted Strategies as a Tool for Designing Green Monomer-Based Molecularly Imprinted Materials

**DOI:** 10.3390/ijms252312912

**Published:** 2024-11-30

**Authors:** Monika Sobiech

**Affiliations:** Department of Organic and Physical Chemistry, Faculty of Pharmacy, Medical University of Warsaw, Banacha 1, 02-097 Warsaw, Poland; monika.sobiech@wum.edu.pl

**Keywords:** molecularly imprinted polymers, computational studies, molecular modeling, polymer simulations, quantum mechanics, molecular dynamics, green chemistry, bio-based materials

## Abstract

Molecularly imprinted polymers (MIPs) are defined as artificial receptors due to their selectivity and specificity. Their advantageous properties compared to biological alternatives have sparked interest among scientists, as detailed in numerous review papers. Currently, there is significant attention on adhering to the principles of green chemistry and environmental protection. In this context, MIP research groups have focused on developing eco-friendly procedures. The application of “greener” monomers and reagents, along with the utilization of computational methodologies for design and property analysis, are two activities that align with the green chemistry principles for molecularly imprinted technology. This review discusses the application of computational methodologies in the preparation of MIPs based on eco-friendly non-acrylic/vinylic monomers and precursors, such as alkoxysilanes, ionic liquids, deep eutectic solvents, bio-based molecules—specifically saccharides, and biomolecules like proteins. It provides a brief introduction to MIP materials, the green aspects of MIP production, and the application of computational simulations. Following this, brief descriptions of the studied monomers, molecular simulation studies of green monomer-based MIPs, and computational strategies are presented. Finally, conclusions and an outlook on the future directions of computational analysis in the production of green imprinted materials are pointed out. To the best of my knowledge, this work is the first to combine these two aspects of MIP green chemistry principles.

## 1. Introduction

Molecularly imprinted polymers (MIPs) are a class of material that exhibit dedicated selectivity and specificity toward selected molecules. These advantageous properties arise from the preparation process, which begins with the formation of a prepolymerization complex between the monomers and the selected molecule, referred to as the template. This complex is formed based on covalent or non-covalent interactions among the ingredients. Next, the structure of the prepolymerization adduct is fixed during the polymerization process, where a polymeric matrix is created from the monomers and cross-linking agent in the presence of a previously bound template. As a result, a polymer network is formed that contains three-dimensional binding sites with the template molecules embedded within. Finally, during the elution process, the template is removed, leaving behind a cross-linked polymeric matrix with binding cavities that are complementary in shape, size, and functionality to the template or similar molecules. Consequently, the resulting product can recognize the desired compound specifically and selectively, comparable to biological enzymes or antibodies. However, unlike biomolecules, MIPs possess valuable properties such as chemical and mechanical stability, resistance to physical factors like temperature or pressure changes, ease of storage and production, and low-cost and straightforward preparation [1,2,3]. Their specificity and advantageous characteristics make MIPs suitable for a wide range of applications [4,5], including separation techniques [6], extraction [7], sensors [8], drug delivery systems [9], cell culture [10], catalysis [11], and synthesis [12].

Despite all the advantages of MIPs, their conventional synthetic processes often rely on non-ecofriendly materials and procedures. Nowadays, significant attention is being paid to the environmental side effects of human activities. At the same time, the advancement of science, industry, and technology is both inevitable and desirable. By combining these two aspects, scientists aim to minimize the negative impacts of human activities [13]. As a result, principles of green chemistry have been introduced in most chemical sciences to reduce the risk of harmful effects on humans and the environment [13,14]. Innovative research on the development of alternative and sustainable green technologies is presented in numerous publications, journals, and conferences, along with many awards and funding programs [13]. Due to the toxicological issues associated with MIP production, particularly concerning the use of harmful and toxic reagents—especially templates and solvents like toluene or benzene—the reliance on large amounts of solvents during washing and elution processes generates significant waste and poses a risk to operators. Consequently, green principles in molecularly imprinted technology have also been explored. Several studies and reviews demonstrate green synthetic strategies for MIPs that align with the principles of green chemistry. The most common changes involve replacing traditional reagents, such as solvents, templates, functional monomers, and washing solvents, with more sustainable alternatives. Additionally, there is an emphasis on applying green polymerization approaches and utilizing eco-friendly strategies such as computational studies, multi-template methods, stimuli-responsive imprinting, and producing self-cleaning MIPs, as well as using modern apparatus [13,15,16,17].

As described, one of the green principles of molecularly imprinted technology is the employment of computational methodology. This technique enables the avoidance of “trial and error” experimental methods during the design and analysis of MIP properties. At the same time, it allows for the rational design of the material by selecting appropriate reagents, their ratios, and synthesis conditions, as well as predicting key properties relevant to adsorption processes and selectivity characterization. The entire process does not require the use of any chemicals or sophisticated apparatus. The application of MIP modeling has increased due to the unlimited access to computing power and various specialized software. Different computational approaches are utilized individually or in combination to simulate MIP systems, such as quantum mechanics (QM), molecular mechanics (MM), or molecular dynamics (MD). The diversity of theoretical methods enables the modeling of various stages and aspects of MIP design, preparation, and analysis. The main directions of using molecular simulations in MIP design related to the analysis of prepolymerization mixtures include: (i) selecting appropriate monomers, cross-linkers, templates, or solvents for synthesis; (ii) determining the appropriate stoichiometry (template-to-monomer or template-to-monomer-to-cross-linker ratio) during polymerization; and (iii) analyzing the effects of various conditions and agents on polymer creation. Additionally, the resulting polymer can be analyzed using computational methods based on (i) structural establishment and optimization, (ii) template–polymer interactions, and (iii) selectivity studies involving the analysis of different analyte–polymer interactions in various solvents. The main parameter analyzed after molecular modeling is the interaction energy, defined according to Equation (1):

∆*E* = *E*_*complex*_ − (*E*_*template*_ + *nE*_*monomer*_) (1)

where ∆*E* is the interaction energy, *E_complex_* is the energy of the analyzed system (monomer-template complex), *E_template_* is the energy of the template, *E_monomer_* is the monomer energy, and *n* is the number of monomer molecules. Many excellent reviews covering the topic of virtual analysis of MIPs have been published in recent years [18,19,20,21,22,23,24,25,26]. However, these studies mainly focus on computational methodology [18,19,26] or the purpose of using computational tools (such as designing the material or analyzing properties and recognition mechanisms) [21,23,24]. Therefore, this description will omit detailed depictions of computational methods and simulation systems and steps.

## 2. Computational Studies on Green Monomer-Based MIPs

The main group of monomers used in molecularly imprinted technology are acrylic or vinyl compounds. Their high affinity for various chemicals and bio-compounds, along with their low cost, accounts for their frequent usage. Additionally, the resulting polyacrylic and polyvinyl polymers are considered non-toxic at concentrations below a few g kg^−1^ [13]. However, many of these compounds may cause a wide range of adverse health effects, from skin or eye irritation to carcinogenic effects [13,27]. Most computational studies of MIP design and analysis predominantly focus on polyacrylic and polyvinyl MIPs [18,19,20,21,23,24].

Based on the properties of the template, the type of imprinting strategy, and the method of polymerization, various classes of chemical compounds can be used to produce MIP materials. In addition to the acrylic and vinyl monomers mentioned, other examples of less toxic, less volatile compounds with better physical properties and a more eco-friendly nature can also be applied in molecularly imprinted technology.

This review presents two aspects of green chemistry principles in molecularly imprinted technology. The first involves the use of “green” functional monomers from various chemical groups, such as alkoxysilanes, ionic liquids (ILs), deep eutectic solvents (DESs), bio-based compounds like polysaccharides, and biomolecules such as peptides. The second aspect pertains to the utilization of computational modeling at various stages of design and property analysis for MIPs constructed from “green” monomers, which differ from traditional organic compounds containing acrylic or vinyl groups. To the best of my knowledge, there are no review publications available that delineate this topic for readers.

### 2.1. Alkoxysilane-Based MIPs

Alkoxysilane functional monomers for MIP production have the general structure of R’_n_–Si–(OR)_4−n_, where R and R’ are organic groups and n can be 0, 1, 2, 3, or 4 [28,29,30,31]. These compounds serve as precursors in the sol-gel process, leading to the formation of polysiloxanes (silicone polymers) that possess beneficial physical and chemical properties [32]. Polysiloxanes are chemically stable, unreactive, and resistant to various environmental conditions, such as water and water vapor, oxygen, and air pollutants. They are gas-permeable, their mechanical properties can be tailored, and they are compatible with body tissue. Moreover, polysiloxanes are flexible, exhibit excellent electrical properties, and have optical transparency. In summary, their properties make them suitable for applications across nearly every major industry [29,33,34]. Silicones are utilized in pharmaceutical and medical fields [35,36], the cosmetic industry [37], electronic manufacturing [38], the automotive and aerospace sectors [39,40], textile products [41,42], construction materials [43], and food processing [44].

The actual and potential commercial utilization of silicones is determined by the specific properties or particular combination of properties associated with the siloxane bond or polysiloxane chain [45]. The structure of silicone polymers, derived from alkoxysilane monomers, features an “inorganic” –Si–O– backbone with “organic” substituents attached to the silicon atom –R_2_Si–O–. The properties and characteristics of the Si–O bond differ from those of the C–O bond. This distinction accounts for the unique properties of polysiloxanes compared to organic polymers containing the –C–O– bond [35,46]. The predicted bond lengths of Si−O and C–O, based on the sum of the covalent radii of the atoms, are 1.77 and 1.42 Å, respectively [47]. Experimental data report values of approximately 1.62 Å for the Si−O bond and 1.43 Å for the C–O bond [35,46]. The calculated R−O−R bond angles in dimethyl ether and hexamethyldimethyl ether are approximately 112° and 128°, respectively, while in their silicon analogs, disiloxane, and hexamethyldisiloxane, these angles are larger: 150.3° and 156.6°, respectively [46,47]. The high Si−O−Si bond angles lower the energy barrier for the rotation of organic groups attached to the Si atom, resulting in substantial flexibility of the polymeric silica chain backbone [46]. The differences between the Si–O and C–O bonds arise from the properties of Si, C, and O atoms, such as their radii (1.77, 0.77, and 0.66 Å for Si, C, and O, respectively) and electronegativities (1.7, 2.5, and 3.5 for Si, C, and O, respectively, on the Pauling scale). The properties of Si and O atoms result in the high polarity of the Si–O bond, along with negative hyperconjugative interactions due to the electron shift from oxygen to silicon. Consequently, the Si–O bond has a partially ionic character, and the O atom is less basic than in the C–O bond [29,47,48,49]. Additionally, the Si–O bond demonstrates a higher bond energy (433–460 kJ mol^−1^) compared to the C–O bond (345 kJ mol^−1^) [46,50,51]. The strong Si–O bond contributes to the chemical and thermal stability of siloxanes, while the large Si−O−Si bond angle facilitates conformational interconversion, reducing surface tension compared to organic polymers [51,52]. Another advantage of polysiloxane chemistry is the ability to use a wide range of organic substituents attached to the Si atom. These organic counterparts influence physicochemical properties such as glass transition temperature, solubility, thermal stability, and surface-free energy [46]. Hydrocarbon side chains with three or more carbon atoms, as well as aromatic groups, reduce the flexibility of the silicone polymer, leading to a higher glass transition temperature [53]. In contrast, methyl groups attached to Si atoms in the polysiloxane chain exhibit remarkable rotational freedom about the Si–O bonds. This results in weak intermolecular interactions and increased distances between polymer chains, leading to a high molar volume and low cohesion energy. Conversely, the rotation of phenyl groups is hindered at lower temperatures and delayed at higher temperatures [54].

All structural characteristics of polysiloxanes, as described, can influence the formation of recognition sites within MIP matrices constructed from alkoxysilane monomer residues. Furthermore, these structural aspects should be considered when evaluating the stability, selectivity, and specificity of MIP binding sites. Siloxane-based MIPs, as well as the use of siloxanes in MIP production, have been previously reviewed [29].

The synthesis of polysiloxanes follows the sol-gel process, which involves hydrolysis and condensation reactions of alkoxysilane monomers [55]. These reactions can occur either sequentially or simultaneously under aqueous basic or acidic conditions [56]. During the synthetic process, different mixtures of oligomers and polymers with diverse structures may form, and various reactions or processes can occur depending on the substrates and conditions [57]. The complexity of the sol-gel synthetic strategy makes computational studies challenging, as they must account for varying conditions and substrate transformations throughout the process. Despite these challenges, numerous computational studies have been conducted to describe the formation of poly- or oligosiloxanes [58,59,60,61,62] and analyze the properties of silanes [63,64], employing methods such as QM/MM, reactive MM, and MD simulations. However, theoretical simulations specifically addressing the formation or properties of silane-based MIPs are less common. The problem could be the existence of monomers in different forms, such as native, hydrolyzed, or condensed, dependent on the conditions. This variability can significantly impact the formation of the prepolymerization complex, a crucial step that determines MIP properties [25]. Nevertheless, some molecular simulation studies of silane-based MIP materials have been performed, using various methods to address aspects such as monomer selection or reagent ratio optimization. Additionally, studies modeling polymer behavior have explored molecular recognition mechanisms. These results have provided valuable insights into the design and functionality of MIPs

The first example of molecular modeling applied to design MIP prepared from alkoxysilane using the sol-gel method was published in 2008 [65]. The paper described one of the most popular applications of computational simulation in the MIP design process: the selection of the most appropriate monomer for synthesis. The authors conducted a comparative study, testing different levels of theory for designing a sol-gel MIP for β-damascenone. 1:1 template-to-monomer complexes were modeled, and nine different monomers were analyzed. The results demonstrated consistency between theoretical (binding energy) and experimental (binding capacity and imprinting factor, IF) studies. Additionally, they indicated that the HF (Hartrree–Fock)/3-21G method, with BSSE (basis set superposition error) correction, may provide a good compromise between theory level and computation time for monomer selection during the sol-gel production of silane-based MIPs. In another example, Mojica and co-workers used different computational methods to optimize and calculate the interaction energies of template–monomer complexes [66]. Systems composed of various components were analyzed, and six different monomers were tested. After comparing interaction energies with experimental results (IF, based on binding capacity), the best correlation was observed for the ab initio (HF) method. In an interesting study, Narula and co-workers screened ten commonly used monomers to estimate their binding affinity toward protein A, used as a template [67]. The polymer was designed for the capture and detection of *Staphylococcus aureus*. The uniqueness of this study stemmed not only from the analysis of silane monomers but also from the analysis of protein as a template and the application of an original theoretical method: molecular docking. Computational studies were performed by docking the template protein with monomers and modeling their intermolecular interactions. The estimated binding energy, the number of distinct conformational clusters, and the number of multi-point interactions were determined. The results identified dopamine as the best monomer in terms of binding energy and silane monomers as superior due to their improved multi-point interactions. Experimental adsorption analysis demonstrated that silane-based MIPs exhibit high binding capacity and IF values, highlighting the importance of interactions with multiple surface residues. In the MIP design process, computational studies also facilitate the selection of the appropriate molar ratio (template-to-monomer-to-cross-linker) of components in the polymerization mixture. MD methodology was introduced to perform a comprehensive theoretical analysis of various interactions occurring in the prepolymerization solution, composed of specific amounts of the template, monomer, and cross-linker molecules [68]. Twenty systems with different numbers of the respective molecules were tested to evaluate the effect of monomer and cross-linker concentrations on template–monomer interactions. Simulation studies helped to identify the most suitable template-to-monomer-to-cross-linker ratio. The reliability of the MD simulations was validated by experimental adsorption results, with a good correlation observed. By enabling reagent and condition selection, computational studies of the prepolymerization complex can be used to analyze interactions among compounds in different forms, depending on reaction conditions. Atta and co-workers investigated the interactions between the monomer unit (tetraethoxysilane, TEOS) in its hydrolyzed form (tetrahydroxysilane) and various template molecules [69]. The authors studied systems consisting of the monomer and template molecule in 1:1 and 2:1 ratios, as well as the disiloxane dimer molecule and the template, using five different templates. The computation results confirmed the data obtained from electrochemical experiments. The normalized current responses of the imprinted molecules were arranged in nearly the same order as the interaction energies.

Apart from the prepolymerization complex analysis, computational studies are applied to analyze the mechanism of molecular recognition at specific binding sites in the MIP matrix. To evaluate the interaction between the template and the imprinted site, Zhu and co-workers calculated the molecular interaction energies between the template and the monomer using the density functional theory (DFT) method [70]. It was indicated that the interaction could be formed via a hydrogen bond between the phenolic hydroxyl group of the template and the amino group of the monomer. Similar studies, which also considered the solvent and pH impact on MIP binding behavior, were presented for 3-aminopropyltriethoxysilane (APTES) as the monomer and 1-naphthyl phosphate as the template [71].

**Figure 1:**
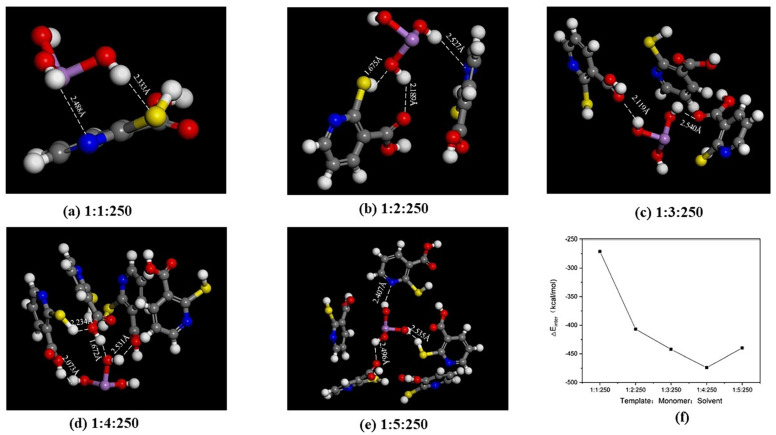
(**a**–**e**) Trajectory snapshots of H_3_AsO_3_ binding with the monomer in dichloromethane at different ratios (the ratios of template:monomer:solvent are indicated). (**f**) Interaction energy of H_3_AsO_3_-monomer in dichloromethane at different ratios. Reprinted from [72] with permission from Elsevier.

Computational modeling studies that analyze multiple aspects—such as monomer, solvent, and ingredient ratio selection, as well as the recognition mechanism of MIPs constructed during the sol-gel process—are scarce. In an excellent study, Wu and co-workers explored the prepolymerization process, cavity recognition, and binding process of H_3_AsO_3_ (the template) on MIPs using QM calculations and MD simulations. 2-Mercaptonicotinic acid was used as the monomer, and the optimal configurations between H_3_AsO_3_ and the monomer in different solvents were analyzed to determine the most accurate solvent and solution concentration. For concentration selection, systems containing different numbers of solvent molecules were tested. Additionally, various template-to-monomer ratios were screened. Trajectory snapshots of H_3_AsO_3_ binding with the monomer in dichloromethane as a solvent at different ratios are shown in Figure 1. Subsequently, the nature of binding interactions between the monomers and the template was determined. Finally, the construction and optimization of the annular cavity model, including the cross-linker, were performed to investigate the dynamic recognition and binding behavior of the MIP to the target. These studies provide insights into the recognition mechanism of imprinted materials, enabling precise regulation of their adsorption performance in real samples [72].

**Figure 2:**
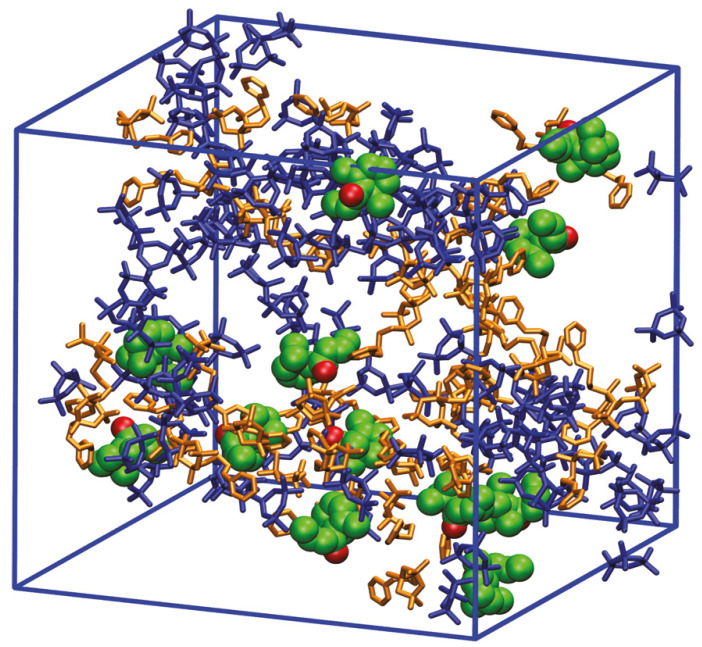
A snapshot of the simulation model (water/methanol 1:3, run 1) shows the template molecules (van der Waals representation in green and red) well dispersed within the box and in close proximity to the 3-(propylaminophenyl)-trimethoxysilane trimer rings. The tetramethoxysilane trimer and 3-(propylaminophenyl)-trimethoxysilane trimer are displayed in blue and gold, respectively. Water and methanol molecules are omitted from the representation. Reprinted with permission from [73]. Copyright 2011 American Chemical Society.

Molecular modeling of the gelation process and the creation of recognition sites in silane-based MIPs presents a more complex challenge due to the computational demands of studying the MIP preparation process. In an interesting series of studies [73,74,75], Azenha and co-workers described an array of MD simulations, where different pre-gelation mixtures representing intermediate stages of the sol-gel process were set up. The primary aim of the research was to estimate the template–gel affinity and template self-aggregation, as well as to study the impact of additional ingredients on MIP formation. The authors used β-damascenone and naproxen in anionic form as templates along with models of trimers constructed from monomeric species: tetramethoxysilane, (3-propylaminophenyl)-trimethoxysilane, and 1-(triethoxysilylpropyl)-3-(trimethoxysilylpropyl)-4,5-dihydroimidazolium iodide, for MD studies. Additionally, the authors tested various molar ratios of methanol–water solvent mixtures, examined different ratios of trimer mixtures to simulate stages of the sol-gel process, considered the formation of ionic forms of the template and trimers due to pH [75], and analyzed the effect of polyethylene glycol on MIP formation [74]. After MD simulation of systems consisting of the template, trimers, solvent, and optionally additional species, analyses of radial distribution functions and interactions were performed. For the polymer dedicated to β-damascenone—a hydrophobic molecule—it was concluded that the presence of the phenylaminopropyl group was critical for binding site formation. Additionally, the use of a methanol-rich mixture as a solvent promoted better homogeneity of the hybrid organic-inorganic MIP and enhanced dispersion of the template. These observations were consistent with experimentally obtained adsorption results [73]. Figure 2 shows a snapshot from the simulation of one of the analyzed models using 3-(propylaminophenyl)-trimethoxysilane trimer. The simulation analysis of systems with polyethylene glycol presence during silica-based MIP formation, designed for β-damascenone sorption, was inconclusive regarding the impact of polyethylene glycol on the association between the template and functional groups [74]. For polymers using naproxen as the template, a strong correlation was found between imprinting-relevant interactions, aggregation, silicate network texturing effects, and the experimentally obtained imprinting effect and porosity features [75].

Despite the considerable number of reports describing silane-based MIPs, papers presenting computational simulation results remain relatively scarce. Table 1 summarizes computational studies, highlighting the tested reagents, methods, and software used in designing and analyzing alkoxysilane-based MIPs. Most of these studies focus on modeling the prepolymerization complex to identify suitable monomers or analyze interactions within the system. Additionally, various analyte/template molecules have been studied, ranging from small ions and small organic molecules to proteins. A significant challenge lies in modeling the polymeric chain and the adsorption process within the recognition cavity to accurately predict MIP properties.

### 2.2. ILs-Based MIP

ILs are another example of green monomers that can be used in the production of MIPs. ILs are often defined as compounds composed entirely of ions, particularly organic cations and organic or inorganic anions, which are molten at or near room temperature, typically below 100 °C [13,87,88,89]. Another definition describes ILs as liquid compounds that exhibit ionic-covalent crystalline structures [90]. The primary ions used for synthesizing ILs include the following: cations (imidazolium, morpholinium, guanidinium, pyrrolidinium, piperidinium, sulfonium, phosphonium, pyridinium, and ammonium); and anions (hexafluorophosphate, dicyanamide, tetrafluoroborate, triflate, acetate, alkyl sulfate, halides, nitrate, metal tri- or tetrachloride, and *p*-methylbenzenesulfonate) [91,92]. Based on the combination of ions in their structure and their specific properties, ILs are classified into several categories, such as task-specific (functionalized), chiral, switchable polarity solvents, bio-ILs, poly-ILs, energetic, neutral, protic, metallic, basic, or supported [92]. ILs possess properties such as low vapor pressure, non-flammability, thermal and chemical stability, high ion density, high ionic conductivity, a wide electrochemical window, and miscibility in water and various organic solvents. Additionally, their eco-friendliness and the tunability of their properties—including density, viscosity, hydrophobicity, polarity, affinity for different compounds, and acid-base properties—through precise cation-anion combinations make them highly attractive for MIP synthesis compared to traditional acrylic and vinyl monomers [13,16,93,94]. The beneficial properties of ILs, along with the wide range of available types, have contributed to their application across various fields, including electrochemistry (metal electrodeposition [95], batteries [96], sensors [97], solar cells [98], fuel cells [99], thermo-electrochemical cells [100], supercapacitors [101]), chemistry (organic reactions [102], nanoparticle synthesis [103], enzymatic synthesis [104]), analytical methods (gas chromatography columns [105], matrices for mass spectrometry [106], stationary phases for high-performance liquid chromatography [107]), additives (lubricating agents [108], corrosion inhibitors [109], surface-active agents [110], plasticizers [111], shale inhibitors [112]), extraction and separation processes (micro-extraction [113], biomass extraction [114], flavonoid extraction [115], wood dissolution [116], water treatment technology [117], enhanced oil recovery [118]), industries [119] (food and bioproduct production [120], biofuel production [121], pharmaceutical production [122]), advanced materials (artificial muscles [123], liquid crystals [124], thermal energy storage devices [125]), environment protection (carbon capture [126], treatment of nuclear waste [127], fuel purification [128], green applications [129]) [130,131]. Due to their unique properties and versatile applications, ILs are of great interest to both scientists and companies. From the perspective of imprinted materials, ILs are particularly attractive because they can form a variety of interactions with different molecules and macromolecules. This is especially important during the creation of specific binding sites in polymeric matrices when ILs are used as functional monomers. Moreover, the obtained MIPs demonstrate impressive performance in aqueous and polar media. Additionally, ILs can be used as solvents/porogens in MIP production due to their impact on the final product’s properties. Other roles of ILs in the MIP preparation process include surface modification/functionalization during the formation of core-shell imprinted materials, crosslinking of the polymeric matrix, or serving as dummy templates. Several excellent reviews have highlighted their potential in MIP fabrication [16,88,89].

Given the growing interest in ILs, computational simulation studies have been employed to analyze IL systems. Various techniques have been used to model IL materials or systems involving ILs, including QM [132], QM/MM [133], and MD [134,135]. Computational studies have focused on interaction analysis within IL systems [132], designing ILs with specific properties and applications [136], or understanding processes occurring in ILs when employed as solvents [137]. Some reviews have discussed the application of computational methods for IL materials [138,139]. Despite the frequent use of ILs in MIP synthesis and numerous descriptions of these processes, the number of reports applying computational studies to IL-based MIPs remains very limited.

Wang and co-workers used the MD simulation method to identify the most appropriate monomer for the MIP preparation process. Simple systems with a monomer-to-template 1:1 molar ratio were simulated, and the binding energy was calculated. Four ILs were tested as candidates for functional monomers. The strongest interaction with the template, pentapeptide thymopentin, was exhibited by 1-vinyl-3-ethyl acetate imidazolium chloride. Using atom transfer radical polymerization, a magnetic MIP was synthesized and demonstrated good adsorption performance toward the template [140]. The same group applied MM and MD methods to identify the template–monomer complex with the lowest binding energy and to analyze interactions within the prepolymerization mixture. In this study, the authors synthesized 1-butyl-3-vinyl imidazolium amino hydrocinnamic acid ([BVIM][Phe]), an IL that functioned as both a functional monomer and a dummy template, to produce MIP microspheres selectively recognizing L-phenylalanine. During the computational analysis, three different systems were tested, each consisting of IL or N-vinylimidazole, the monomer (4-vinylpyridine), and the template in a molar ratio of 1:2:1. The first system, which showed the lowest binding energy, included the previously synthesized [BVIM][Phe] acting as both a monomer and the template, along with two molecules of 4-vinylpyridine. The other two systems were composed of [BVIM]Br, two molecules of 4-vinylpyridine, and the template (L-phenylalanine), and N-vinylimidazole, two molecules of 4-vinylpyridine, and the template (L-phenylalanine). The synthesized [BVIM][Phe] formed electrostatic interactions within the template–monomer complex, resulting in the creation of stable and complementary imprinting cavities through vinyl-imidazolium polymerization. The computational results were confirmed by adsorption and selectivity experimental tests of MIP microspheres obtained through the surface-initiated reversible addition−fragmentation chain-transfer method [141,142]. Zhao and co-workers performed MD simulations of the prepolymerization complex, first, to identify the best monomer and second, to determine the optimal template-to-monomer molar ratio for preparing MIP specific to gastrodin. The authors included solvent (ethanol) molecules in the MD simulation box during the calculations. Five different monomers—acrylic acid, methacrylic acid, dehydroabietic acid [2-(acryloyloxy)ethyl] ester, 3-(2-carboxyethyl)-1-vinylimidazolium bromide IL, and 1-vinyl-3-tetradecylimidazolium bromide IL—were tested. Among these, the system with a 1:1 template-to-monomer molar ratio showed the lowest binding energy for 1-vinyl-3-tetradecylimidazolium bromide IL. In the next step, complexes with different molar ratios of the best monomer to the template were analyzed, with the most favorable result obtained for a 4:1 ratio. Additionally, the screening of functional monomers was further validated using ultraviolet and visible spectrophotometry, which aligned with the computer simulation results [143]. In another study, Chen and co-workers employed DFT computational analysis to examine interactions between the functional monomer (1-carboxymethyl-3-vinylimidazolium bromide) and the template molecule (chlortetracycline). To identify the binding sites and the types of forces between the monomer and the template, independent gradient model analysis was performed on the complex systems. The results revealed that the main interaction sites were the carboxyl and hydroxyl groups, while the primary forces were hydrogen bonding and π-π stacking [144]. The DFT method was also employed by Das and co-workers in a comprehensive theoretical study aimed at designing IL-MIP composite with graphene oxide. The study included the selection of an appropriate IL functional monomer for the template, 4-hydroxybenzoic acid, as well as geometry optimization and frequency calculations for template–monomer complexes and template–monomer–graphene oxide complexes with selected ILs. Additionally, calculated and experimental infrared (IR) spectra were generated for the selected ILs and their template–monomer complexes. To validate the theoretical results, the chosen MIPs were synthesized, and rebinding studies were conducted. The authors tested 17 different IL functional monomers, calculated the Gibbs free energy for template–monomer complexes, and analyzed the interactions within the studied systems. For two monomers characterized by the lowest and medium Gibbs energy values and for their template–monomer complexes, IR spectra were both calculated and experimentally measured. Furthermore, for four selected systems with varying ILs and different Gibbs energy values, molecular modeling was extended to include graphene oxide moieties, providing an in-depth analysis of the interactions. The experimental rebinding studies demonstrated that the binding capacity trends closely matched the simulation results [145]. This work is a rare example of support (graphene oxide) modeling incorporated into MIP computational studies.

In summary, computational studies on IL-MIP systems are exceedingly rare, with most research focusing exclusively on interactions within prepolymerization complexes. While some studies have moved beyond the simplest 1:1 template-to-monomer systems to include additional monomer or solvent molecules, there is a notable lack of work modeling polymeric chains or exploring roles for ILs beyond their use as monomers, such as cross-linkers or solvents. The ionic structure of ILs and the multitude of potential electrostatic interactions likely present significant challenges for such studies.

### 2.3. DES-Based MIPs

Despite the advantages of ILs, there are concerns regarding their toxicity and biodegradability [16]. To address these issues, another class of compounds with similar properties, called DESs, was developed. DESs represent a relatively new class of materials, with the first paper about them published in 2001 [146]. While DESs are sometimes considered a subclass of ILs due to their shared characteristics, they are often treated as a distinct group [147]. DESs possess many of the attractive properties of ILs but are additionally inexpensive, biodegradable, non-toxic, and easier to synthesize [148]. Whereas ILs are composed of cations and anions, DESs consist of various hydrogen bond acceptors and hydrogen bond donors. Based on the type of complexing agent used, DESs are classified into five groups: type I, consisting of quaternary ammonium salts and metal chlorides; type II, combining quaternary ammonium salts and metal chloride hydrates; type III, built from quaternary ammonium salts and hydrogen bonds donors (typically organic residues such as amides, carboxylic acids, or polyols); type IV, including metal chloride hydrates and hydrogen bond donors; and the new type V, composed of non-ionic molecular hydrogen bond donors and acceptors. The main hydrogen bond acceptors in DES production include choline chloride, choline fluoride, N-ethyl-2-hydroxy-N,N-dimethylethanaminium chloride, 2-(chlorocarbonyloxy)-N,N,N-trimethylethanaminium chloride, N-benzyl-2-hydroxy-N,N-dimethylethanaminium chloride, tetra-n-butylammonium bromide, tetra-n-ethylammonium chloride, tetra-n-methylammonium chloride, betaine benzyltriphenylphosphonium chloride, methyl triphenylphosphonium bromide, lidocaine, alanine, glycine, proline, histidine, nicotinic acid. Common hydrogen bond donors include urea, acetamide, 1-methylurea, 1,3-dimethylurea, 1,1-dimethylurea, thiourea, benzamide, glycerol, ethylene glycol, triethylene glycol, 1,4-butanediol, phenol, 1-naphtol, menthol, thymol, malonic acid, benzoic acid, adipic acid, oxalic acid, succinic acid, decanoic acid, dodecanoic acid, citric acid, lactic acid, glucose, and fructose [148,149]. Due to their similar properties to ILs, DESs have comparable areas of application [148,150]. Their eco-friendly and advantageous properties make DESs highly attractive for use in MIP synthesis, where they can serve various roles, such as monomer, solvent, cross-linker, modifier, or template. Their application in MIP production has been highlighted in several excellent reviews [17,151,152].

As with ILs, different computational studies have been conducted on DESs. Identifying the first simulation studies is challenging, as these were performed when DESs were initially classified as protic ILs [148,153]. QM methods are used in DES modeling to elucidate their physical, thermodynamic, and structural associations, particularly the relationship between melting point depression and solvent organization influenced by hydrogen bond concentration [154,155]. MD methods are better suited for simulating large systems, enabling the examination of DES organization as well as their thermodynamic and transport properties [154,156]. Several excellent reviews summarize the application potential of computational methods for studying DES materials [153,157,158]. However, despite the widespread use of DESs in MIP preparation and the popularity of computational studies on DESs, the number of publications addressing computational studies related to DES-based MIPs remains very limited.

Li and Row synthesized DES from choline chloride and methacrylic acid, utilizing it as an eco-friendly surfactant and functional monomer in the preparation of levofloxacin-imprinted nanoparticles. The DES production process and its free energy of formation were computationally evaluated using the DFT method. The most stable DES structure, consisting of one methacrylic acid molecule and two choline hydrochloride units, was identified and subsequently applied in the preparation of MIP nanoparticles [159]. In a related study, the same authors used the DFT method to investigate MIP formation and to analyze and model interactions between the DES functional monomer, the template, and the support material—hexagonal boron nitride—during the synthesis of MIP designed for the extraction of flavonoids (quercetin, isorhamnetin, and kaempferol) from *Ginkgo biloba* leaves. The DES was composed of choline hydrochloride, ethylene glycol, and caffeic acid. Caffeic acid facilitated interactions with the support via its –OH groups and interacted with the template and analytes through its –COOH group. Electrostatic potential maps of the functional monomer and analytes were examined to identify potential interaction regions. Modeling of 1:1 caffeic acid–template (quercetin)/analyte complexes revealed that hydrogen bonds were the primary interactions responsible for analyte recognition. Similar energy values for caffeic acid–analyte complexes across different targets suggested comparable selectivity of the MIP for all analyzed compounds. In a model comprising four caffeic acid molecules and one template, seven stable intermolecular hydrogen bonds between quercetin and monomers were observed, demonstrating the complex’s stability. Results from extraction studies validated the theoretical predictions [160]. In another notable study, a broader computational analysis was performed to model and analyze the properties of MIP at the molecular level and to understand the binding mechanism between the template/analyte (metronidazole), DES (composed of choline chloride, ethylene glycol, and methacrylic acid in a molar ratio of 6:6:2), and cross-linker (ethylene glycol dimethacrylate, EGDMA). Interactions among MIP components were evaluated in various models representing single-ring (involving one template and DES unit) and double-ring (involving two templates and two DES units) MIPs, optimized using the DFT method. The study also examined the influence of varying amounts of cross-linker. The interaction energy calculations revealed that the double-ring MIP type was more stable than the single-ring model. It was determined that hydrogen bonding among the template, DES, and EGDMA played a critical role in the imprinting process of the most stable system consisting of two metronidazole molecules, two DES units, and eleven EGDMA molecules. Various parameters describing the chemical properties of the most stable complex formation were calculated, offering insights into the stability of the system. Electrostatic potential maps of MIP components were analyzed to identify reactive sites. Theoretical analysis of a control polymer highlighted three types of binding cavities compared to one cavity type found in the MIP. Simulation-based selectivity studies involving three antimicrobial agents demonstrated that the MIP exhibited specificity toward metronidazole. Computational findings were experimentally validated, confirming the in silico predictions [161].

To summarize, DES-MIP computational simulation studies remain scarce, but they provide valuable insights into the preparation and recognition mechanism of these systems. While different aspects of DES-MIPs, including binding interactions and stability, have been explored, there are no reports addressing DES-MIP polymeric chain modeling or the production process with DES as a cross-linker. The multiplicity of molecules when DES is the polymeric mixture ingredient and the probability of large amounts of interactions likely presents a significant challenge to computational modeling.

### 2.4. Bio-Based and Biomolecule-Based MIPs

Among the available MIP precursors, bio-based materials and biomolecules stand out as the safest and greenest options. The term “bio-based” refers to materials consisting of ingredients derived from natural resources, such as plants or animals. This category includes a variety of molecules, such as poly- and oligosaccharides like chitosan, cellulose, cyclodextrins, alginate, or amylose. Additionally, biomolecules such as amino acids and protein macromolecules are used in MIP synthesis. These bio-based precursors offer advantageous properties, including biocompatibility, biodegradability, renewability, eco-friendliness, cost-effectiveness, carbon neutrality, and multifunctionality. These attributes make them highly attractive for MIP preparation and suitable for applications across a wide range of industrial sectors [13,162,163]. In recent years, significant efforts have been directed toward the development of sustainable materials and processes that align with the principles of green chemistry. The goal is to reduce waste and environmental impact while advancing technologies that improve human living standards. As a result, polysaccharides and proteins have become increasingly prominent in advanced industries [164]. Various reviews have highlighted the diverse application areas of bio-based materials [164,165,166,167,168,169]. Additionally, the use of specific bio-based materials like chitosan or cyclodextrin in MIP preparation has been reviewed in recent high-quality papers [170,171].

Polysaccharide materials and MIPs are frequently studied using QM and MD computational methods. QM methods have been applied for chitosan- [172], cellulose- [173], alginate- [174], cyclodextrin- [175], and amylose-based [176] materials to predict chemical structures, spatial conformations, conformational changes, molecular interactions, or to design materials with desired properties. Numerous MD simulation studies have also been reported for these materials [177,178,179,180,181], providing valuable insights into the atomic-level details of the studied systems and explaining properties observed in experimental analyses. Molecular modeling studies employing various computational methods are also commonly used to analyze protein-based materials and have been discussed in previously published reviews [182,183]. Nevertheless, biomaterial-based MIPs are rarely analyzed through computational simulation methods, even though their preparation, analysis, and applications are frequently described.

Chitosan, a linear polysaccharide obtained through deacetylation of chitin, consists of β(1-4) linked 2-amino-2-deoxy-β-D-glucopyranose and 2-acetamino-2-deoxy-β-D-glucopyranose units and can be easily modified [170]. Due to its biocompatibility and the presence of amine and hydroxyl functional groups, structural modification and cross-linking of chitosan are possible during the MIP preparation process when the polysaccharide is used as a functional monomer or as a supporting matrix [184]. Rahangdale and Kumar employed DFT calculations to support the use of a dummy template (4-hydroxybenzoic acid) instead of the target molecule (salicylic acid) for the synthesis of modified chitosan-based MIPs [185,186]. They also identified the best grafting agent for the functionalization of chitosan for the production of MIP recognizing Cd ions [187] and demonstrated the formation of stable prepolymerization complexes when acrylamide-grafted chitosan was used instead of unmodified chitosan. The stability of these prepolymerization complexes, formed between the template or dummy template and either chitosan or modified chitosan, was evaluated based on changes in Gibbs free energy. Simple 1:1 monomer-to-template molar ratio models of the complexes were used, with chitosan modeled as a single molecule of 2-amino-2-deoxy-β-D-glucopyranose. Energy and interaction analyses validated the hypothesis regarding the suitability of the dummy template and grafted chitosan for MIP synthesis [185]. In another study, Wang and co-workers employed DFT calculations to design high-performance ion-imprinted chitosan microspheres. Chitosan was selected as a functional monomer to interact with Cu(II) ions, which were used as a template. The authors determined the most suitable template-to-monomer ratio by modeling systems with 1:2, 1:3, and 1:4 molar ratios, corresponding to the bridge, triangle, and central models, respectively. Chitosan was modeled as a single molecule of 2-amino-2-deoxy-β-D-glucopyranose. The 1:2 system, in which the template interacted with both the amino and hydroxy groups of the monomer, exhibited the best adsorption energy. Additionally, DFT simulations and HOMO-LUMO orbital analyses were conducted to study the adsorption and orbital energy values in complexes where Cu(II) ions were replaced by other ions, including Ca(II), Co(II), and Mn(II). The computational results confirmed experimental adsorption outcomes, showing that the polymer had the strongest affinity for Cu(II) ions [188]. In another interesting example, bifunctional monomer oligomer-based composite molecularly imprinted membranes for the electrochemical monitoring of Sudan I were designed with the support of computational methods. Quantum chemical calculations were performed during the MIP design process to evaluate the conjugation between the template and functional monomers by measuring the electronic stabilization energy of template–monomer complexes. Neutral amino acids and glucosamine were used to construct the 4-pentenoyl-aminoacyl-glucosamine or 4-lipoyl-aminoacyl-glucosamine functional monomers. Stabilization energy values for template–monomer (1:1 molar ratio) systems were analyzed to identify the most suitable monomer for synthesis. The synthetic process for the electrochemical sensor was then successfully carried out in two stages: (1) self-assembly of a pentenyl (lipoic acyl)-isoleucyl-chitosan oligosaccharide layer on an Au nanoparticle-modified glassy carbon electrode, and (2) polymerization of the molecularly imprinted membrane using Sudan I as the template, pentenyl-asparaginyl-chitosan oligosaccharide as the monomer, and EGDMA as the cross-linker [189].

Another polysaccharide used for MIP preparation, supported by computational modeling, is sodium alginate—a linear polysaccharide produced by brown algae or microbial culture. It consists of (1-4) linked α-L-guluronic acid and β-D-mannuronic acid, arranged in heterogenous or homogenous blocks capable of forming gels. The diverse arrangement of molecules in alginate results in a wide variety of structures, molecular weights, and physicochemical properties [167,190]. Due to its advantageous properties, typical of bio-based materials, along with the simplicity of cross-linking with CaCl_2_ and the potential for producing alginate-based MIPs for protein recognition, sodium alginate is frequently used in MIP production as a monomer or supportive matrix. Gao and co-workers employed DFT calculations to elucidate the effects of ion imprinting, determine the binding configurations of [PdCl_4_]^2−^ or AlCl_4_^−^, and study the binding energy and electron migration in complexes formed after Pd(II) or Au(III) adsorption. The thiourea-grafted sodium alginate model was represented as a dimer composed of thiourea modified with α-L-guluronic acid and β-D-mannuronic acid, and its binding configuration was determined. The models included one molecule of [PdCl_4_]^2−^ or AlCl_4_^−^ and one molecule of the sodium alginate dimer, with the solvent incorporated using the conductor-like screening model (COSMO). The interactions between the template and polymer, as well as binding energies, were analyzed and calculated. The theoretical predictions were consistent with experimental characterization analyses [191,192].

The next class of bio-based compounds is cyclodextrins—cyclic oligosaccharides with unique structural properties. Commonly used cyclodextrins are natural and occur in three forms: α, β, and γ, containing six, seven, or eight α-D-glucopyranose units linked by α(1-4) glucosidic bonds and are obtained from the enzymatic degradation of starch. They have a cylindric shape with a hydrophobic cavity and a hydrophilic exterior surface, allowing them to form host–guest inclusion complexes through various types of interactions. Due to their properties typical of bio-based materials and the ability to create multiple molecular interactions with the guest, cyclodextrins are frequently applied in MIP creation, enhancing selectivity [171,193,194]. Liu and co-workers applied the DFT method to identify the best monomer for preparing β-cyclodextrin supramolecular imprinted polymer fibers for the selective detection of parabens. The authors analyzed a simple template (propylparaben):monomer 1:1 complexes and calculated binding energy values. Five different cyclodextrin derivatives and three commonly used functional monomers were computationally studied, and the results of extraction experiments successfully verified the theoretical findings [195].

Amino acids are used as functional monomers for MIP preparation [196], especially in the development of electrochemical sensors [197]. However, computational studies dedicated to amino acid-based MIPs are very rare. Salajegheh and co-workers employed the DFT method to identify the best monomer [198] and the template-to-monomer molar ratio [198,199] during the construction of electrodes for sensing morphine [198] and theophylline [199]. Arginine, lysine, and phenylalanine were tested as monomers, and different template-to-monomer ratios (ranging from 1:1 to 1:5) were analyzed. Binding energy values were calculated, solvation effects were considered using the polarizable continuum model (PCM), and the possibility of hydrogen bond formation in prepolymerization complexes was examined. The results indicated that the best template-to-monomer ratio was 1:4, and arginine was identified as the most effective monomer for theophylline sensor. Based on theoretical predictions, a molecularly imprinted poly-L-lysine/sodium alginate-activated carbon/glassy carbon electrode for morphine and molecularly imprinted polyarginine/sodium alginate-multi walled carbon nanotubes/glassy carbon electrode for theophylline sensing were successfully constructed and experimentally validated. In another interesting example, Roy and co-workers applied a similar computational methodology to design a metronidazole-probe sensor based on an imprinted biocompatible nanofilm for rapid and sensitive detection of anaerobic protozoan [200]. The authors tested ten compounds as potential functional monomers, including conventional monomers and amino acids, analyzed different template-to-monomer ratios (ranging from 1:1 to 1:3) for all compounds, and selected the most suitable solvent for the polymerization process from four candidates. Finally, glutamic acid and water were chosen for sensor preparation based on the computed binding energy values of the various systems and porogens.

**Figure 3:**
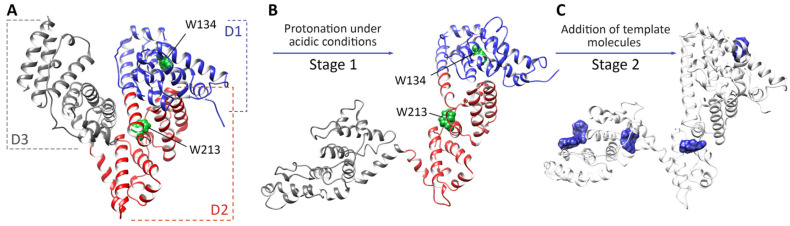
The molecular structure of BSA at different stages of imprinted protein synthesis: (**A**)—The native structure corresponds to PDB ID 4F5S; the three main domains are shown in blue, red, and gray, respectively; tryptophan residues are shown in green. (**B**)—Protein protonation is associated with the event of new conformation formation. (**C**)—BSA–zearalenone complex: template molecules forming a series of intermolecular contacts with new conformation of protein are shown. Used with permission of Royal Society of Chemistry, from [202]; permission conveyed through Copyright Clearance Center, Inc.

Peptides and proteins are also employed in the creation of green molecularly imprinted materials, but theoretical simulations of prepolymerization systems or resulting products are rarely encountered. In an interesting example, Battista and co-workers applied the DFT method not to find a monomer or design the most appropriate polymer but to interpret the effect of the template (bovine serum albumin, BSA) protein on the fluorescence of the peptide multi-functional block, which served as an active assistant recognition element (AARE). The authors prepared a prepolymerization complex in which the AARE—dansyl-modified serum albumin peptide—specifically interacted with the template, the BSA protein (peptide/protein supramolecular assembly). The adduct was then co-polymerized with acrylate monomers while retaining the fluorophore (dansyl) in an appropriate location to produce a hybrid peptide-polymer imprint. The ground and excited state potential energy surfaces of a dansyl-peptide model system were investigated, with solvent effects described using the PCM model. The emission energy of the dansyl molecule was calculated in two solvents: first, non-polar cyclohexane, to simulate the effect of protein polarity on the dansyl emission energy, and second, water, to simulate the local environment in the absence of protein. The calculated values were consistent with experimental results obtained from spectrophotometric titration with the BSA protein [201]. The second example of the application of computational studies in designing, property analysis, and explaining the molecular recognition mechanism of protein-based imprinted materials was described by Ilicheva and co-workers, utilizing foodborne toxins (zearalenone, deoxynivalenol, aflatoxin B1, and kwakhurin) as templates. In particular, the authors simulated the first two stages of imprinted protein synthesis to demonstrate how synthesis conditions could affect structural changes in the protein matrix as well as template–protein interactions (Figure 3). Additionally, a discussion about the effectiveness of contemporary molecular modeling methods in explaining the mechanism behind the imprinting of proteins was provided. All-atom MD simulation results of the first stage of imprinting protein preparation revealed that BSA was characterized by a high positive charge under acidic conditions before template addition. This observation indicated that electrostatic interactions induced protein conformational rearrangement, resulting in the appearance of additional template-binding regions. Consequently, the sorption capacity and affinity of BSA toward the template increased. Computational studies simulating the second state of imprinted protein synthesis were performed using blind docking and post-docking MD simulations to identify the binding sites on the protein surface and determine the most stable recognition sites. Results from these simulations helped to select the optimal initial template-to-protein molar ratio for synthesis and analyze interactions within the template–protein system. Systems with various template molecules were tested, including those with dual templates [202].

To sum up, the reported number of computational studies on biomaterial-based or biomolecule-based MIPs is very scarce compared to the numerous reports describing the preparation, analysis, and application of such materials. One challenge for simulation is the size of the MIP precursor, which is a large macromolecule, unlike small acrylic or vinyl monomers. This larger size is associated with more complex interaction patterns within the macromolecule itself and between the precursor/MIP and the template/analyte. This complexity necessitates the use of methods such as molecular docking in addition to QM techniques, which are more commonly applied to smaller molecules. Furthermore, the conformational changes of saccharides or proteins present an additional challenge during computational simulations, making MD studies essential to identify optimal conditions such as solvent, pH, or temperature for the synthetic process. These operations require substantial computational power and resources.

## 3. Computational Strategies in Green Monomer-Based MIP Simulations

The description of computational studies applied to green monomer-based MIPs reveals that the selection of computational methodology depends on the aim of the simulation and the size of the system. QM methods, such as ab initio (mainly HF), DFT, or semi-empirical approaches, are primarily employed for geometry optimization, calculation of complexation or binding energy, thermodynamics of complex formation, determination of the optimal template-to-monomer ratio, and HOMO-LUMO analysis [18]. These methods describe the electronic structure of the analyzed system, providing better insights into the non-covalent interactions present in the prepolymerization system [203]. QM methods are suitable for smaller systems, where they can simulate template–monomer interactions during monomer screening to select the optimal components for synthesis or to explain template/analyte-monomer interactions during molecular recognition. However, increasing the number of atoms in the system requires extensive computational resources and time [24]. For designing MIPs constructed from green monomers using the ab initio approach, the HF method with basis sets such as 3-21G, 3-21+G, 3-21+G*(H), 6-31G(d), SVP, or SV(P) has been applied yielding results that are consistent with experimental analyses [65,66,76,79,86,189]. Comparative studies between ab initio and semi-empirical methods, such as AM1 or PM3 (which are faster than ab initio), have concluded that ab initio methods generally produce better results [65]. For semi-empirical methods, the best results are obtained when all species present in the system are included [66]. The choice of the basis set is critical for ensuring reliable results but must be optimized to balance accuracy with computational efficiency [66]. Additionally, incorporating solvent effects during calculations using PCM or COSMO models provides more accurate results. Including the BSSE correction is essential during calculations [76]; however, when small basis sets are used, BSSE corrections are less significant because the superposition of the basis set is too small. An interesting alternative to commonly used ab initio and semi-empirical methods is the composite HF-3c method proposed by Grimme in 2013. Positioned between semi-empirical and DFT methods, it is based on the HF method with the MINIX basis set (a minimal Gaussian-type basis set). The HF-3c method accounts for dispersion interactions, computes interatomic distances, and is free from BSSE due to the limited basis set size, achieved through three atom-pairwise semi-empirical corrections. Additionally, it can calculate the electronic structure of both small organic molecules and larger systems. The method has been tested, showing promising results in predicting the geometries of small organic molecules, interaction energies, and the geometries of non-covalently bound complexes, supramolecular systems, protein structures, and in the mechanical stability screening of microporous materials [204,205].

The most commonly used QM method in MIP calculation studies is DFT, characterized by an excellent price-to-performance ratio among QM methods. The majority of template–monomer interaction calculations are conducted using this approach. For MIPs constructed from green monomers, DFT is also the most popular method. In particular, B3LYP is frequently employed for calculating binding energies in green monomer-based MIPs [203]. Accurate results have been obtained using basis sets such as 3-21G, 6-31G, 6-31G(d), 6-31G (d,p), 6-311G(d,p), 6-31+G, 6-311+G(d), 6-311+G(d,p), and 6-311+G(2d,2p) [77,79,80,86,144,145,185,187,188,200]. Other DFT methods found in the literature for modeling green MIPs include LC-WPBE/6-31G(d,p) [82], M06-2x/6-311++G(d,p) [71], and GGA/BLYP double numerical plus polarization set, equivalent to 6-31G** [191,192]. Additionally, BSSE corrections and solvation models are often applied to ensure calculation accuracy. For instance, Grimme’s D3BJ dispersion correction has been utilized in one example [144]. Some promising alternatives to the traditional DFT methods are emerging composite methods, which are particularly efficient for systems with a large number of atoms. These methods incorporate appropriate weak interaction potential models, including dispersion-type interactions, significantly improving energy descriptions. Excellent examples of emerging low-cost electronic structure methods include PBEh-3c (Perdew–Burke–Ernzerhoff threefold corrected) and *r*^2^SCAN-3c. PBEh-3c, following the HF-3c method, represents another “low-cost” approach suitable for computing structures and electronic energies in large chemical systems. This method is free of BSSE and accounts for most interactions in a physically sound and asymptotically correct manner. Additionally, it yields reliable results for non-covalent interaction energy calculations in both small and large complexes. PBEh-3c employs a polarized valence double-zeta basis set, which enhances the energetic description without sacrificing computational efficiency and includes two atom-pairwise corrections [206]. The newly described *r*^2^SCAN-3c method, belonging to the “3c” family, uses the mTZVPP basis set (valence triple-zeta with two sets of polarization functions, a tailor-made triple-zeta Gaussian basis set), a charge-dependent semi-classical D4 dispersion model, and gCP correction for London-dispersion and BSSE. It is based on the *r*^2^SCAN meta-generalized gradient approximation. The performance of *r*^2^SCAN-3c has been evaluated against the GMTKN55 database, which covers general main group thermochemistry, kinetics, and non-covalent interactions [207]. Additional benchmarks have demonstrated its effectiveness for non-covalent interactions, organometallic reactions, lattice energies, and adsorption on polar and non-polar surfaces [208].

When the size of the simulated system is large and multi-molecule models are constructed, atomistic simulation methods, such as MM and MD, are commonly employed. These methods are less computationally intensive and time-consuming compared to QM approaches, but they also have lower accuracy. MM uses classical mechanics and describes energy due to bond length, angle, dihedral angle, and non-covalent interactions. MD, on the other hand, numerically solves Newton’s equations of motion to simulate the time evolution of configurations of interacting atoms. MM is typically used for geometry optimization of large systems, docking studies, and complex formation or binding affinity evaluations, while MD is employed to analyze conformational energies, non-covalent interactions, and interatomic distances over time using numerous methods [18,203]. For simulations, force fields, which describe the forces between atoms, are critical. In the molecular modeling of green MIPs, MM methods are rarely used independently and are typically combined with other methods, such as QM approaches. Notable examples of force fields used in such calculations include MMFF94, which has been applied to search for initial conformation of template–monomer complexes prior to QM calculations [79], and COMPASS, which has been utilized to analyze template–monomer interactions in smaller systems [141]. These force fields have been shown to provide reasonable and reliable results in their respective applications.

During MD simulations, various aspects of green MIP design, preparation, and analysis are studied. These include template–monomer interactions [142], interactions between different components of the simulated systems such as template, monomer, cross-linker, and solvent [86], the atomistic basis of gel matrix–template interaction and conformational changes [76], the prepolymerization process, cavity recognition, and the binding process [72], as well as protein–template interaction changes during MIP synthesis [202]. Force fields employed in these computational studies that provide satisfactory results include COMPASS [142], OPLS-AA [73,202], AMBER [67], and UFF [72]. The majority of MM and MD applications in green MIP design, analysis, and evaluation have been described for silica-based MIPs. In contrast, very few studies have applied these methods to MIPs derived from other green monomers. Notably, no examples of atomistic simulations were found for saccharide-based MIPs. In summary, MM and MD simulations of such MIP systems are underexplored, and their application needs further study and evaluation due to their current rarity in this field.

## 4. Conclusions and Outlook

The review provides an extensive overview of the use of computational methods in the design and analysis of green monomer-based MIPs. Simulation techniques have been applied to study imprinted materials made from non-acrylic/vinylic monomers, such as alkoxysilanes, ionic liquids, deep eutectic solvents, bio-based precursors (e.g., saccharides like chitosan, sodium alginate, cyclodextrins), and biomolecule-based precursors (e.g., peptides and proteins) that offer advantages from a green chemistry perspective. The use of green precursors in MIP preparation, alongside computational methods in the MIP design process, aligns with green chemistry principles defined for molecularly imprinted technology [13]. These strategies are applied to create eco-friendly, biocompatible molecularly imprinted materials.

The advancement of computational methods, as well as the availability of diverse software packages and computing resources, has expanded their application to produce and analyze MIPs made from more complex reagents than traditional acrylic or vinylic monomers under a variety of conditions. However, despite the common use of simulation techniques, such as QM, MM, MD, or their combination in acrylate/vinyl-based MIP formation and in non-MIP polymers made from green monomers, computational studies specifically addressing green MIP production remain limited. This scarcity underscores the need for continued research and development of computational approaches that facilitate the modeling of sophisticated MIP materials. This review addresses this gap by summarizing recent studies on computational strategies in green MIP preparation.

Researchers face many challenges in computational studies of new materials. The size of the simulation systems, the diversity of molecules, and the large number of interactions in prepolymerization systems necessitate precise method selection or the development of new techniques. In this context, alternative computational strategies, such as Monte Carlo simulations or metadynamics, could be beneficial. Additionally, artificial intelligence and machine learning, which are rapidly advancing, may offer valuable tools in the MIP design process. Initial attempts to apply machine learning for MIP design and analysis, as well as the potential of this approach, have been highlighted in an excellent review [209]. An intriguing study described the computational modeling of APTES as a monomer and TEOS as a cross-linker for MIP design, employing a random forest machine-learning algorithm. This approach facilitated the development of a dual-emission MIP-based sensor for the rapid detection of pretilachlor [210]. Furthermore, the application of machine learning in modeling green solvents such as DESs and ILs has been reviewed [211], offering valuable insights for designing DES- or IL-based MIPs using the aforementioned computational method. Achieving a balance among simulation time, cost, resource availability, and result accuracy is also crucial.

Structural flexibility in green MIPs, along with macromolecular precursors and conformational changes influenced by variables such as pH, presents further challenges in molecular simulations. To address these demands, advanced modeling protocols should be developed. Rapidly evolving computational methods, software, and resources offer promising tools for green MIP production, making the process more eco-friendly—not only through the use of environmentally friendly reagents but also by applying a green design strategy.

## Figures and Tables

**Table 1:** Summary of computational studies highlighting the tested reagents, methods, and software used in designing and analyzing alkoxysilane-based MIPs.

Simulation Purpose and Result	Monomer Used in Synthesis/Other Tested Cross-Linker (*CS*) Other Components in Simulations	Solvent Used in Synthesis/Tested	Template	Computational Methods	Software	System Modelled and Calculations	Ref.
Monomer screening— choice the optimal	TMOS, C_8_TMOS, C_6_F_5_TEOS, PhNHTMOS/NH_2_PhTMOS, PhTMOS, NH_2_TMOS, SHTMOS, COOTMOS *CS*: TMOS	Methanol/water, acetone, dichloromethane, THF	β-damascenone	MM/MM2 FF—conformational search; HF 3-21G, then HF/3-21+G, HF/3-21+G*(H), B3LYP/3-21G (HF/DFT hybrid), HF-PCM/3-21+G*(H)—geometry optimization; AM1, PM3—minimum energy location; SCFMI—correction of BSSE; PCM—solvation model	GAMESS	Prepolymerization complex as systems with template:monomer ratio 1:1 in solvent; Interaction energy calculated	[65]
	ATEOS/PhTEOS, CNETMOS, C_2_TMOS, C_5_TEOS, C_8_TEOS *CS*: TEOS, TMOS *End capping agent*: TMCS	NA/acetonitrile, water, methanol, ethanol, THF	Tetracycline	PM3—conformational search of molecules, geometry optimization; HF 3-21G, 6-31G(d), SV(P), SVP—geometry optimization; MMFF94—conformational search of complexes; B3LYP/3-21G—checking that comparable structures were obtained; AM1—optimization of the complex; COSMO—solvation model Tests of different methods	Spartan, Gaussian	Prepolymerization complexes as systems with template:monomer ratio 1:1; template:monomer:CS ratio 1:1:1; template:monomer:CS:TMCS 1:1:1:1 in solvent; Interaction energy calculated	[66]
	ATEOS, PhTEOS, CNETMOS, C_2_TMOS, C_4_TMOS, C_5_TEOS, C_8_TEOS, TEOS, TMOS *CS*: TEOS, TMOS *End capping agent*: TMCS	Ethanol/-	Tetracycline	PM3—conformational search of molecules, geometry optimization; HF 3-21G, SV(P), SVP—geometry optimization; MMFF94—conformational search of complexes; B3LYP/3-21G—checking that comparable structures were obtained; BSSE correction	Spartan, Gaussian	Prepolymerization complexes as systems with template:monomer ratio 1:1; Interaction energy calculated	[76]
	Dopamine, PhTEOS, UPTMOS, TEOS, APTES/MAA, NIPAM, MA, APBA, AA *CS*: TEOS *Carrier*: Fe_3_O_4_@SiO_2_ with APTES and GTA	Phosphate buffer/-	protein A	Molecular docking, AMBER	Autodock	Prepolymerization complexes as systems with template:monomer ratio 1:1; Interaction energy calculated	[67]
	PAPhTMOS, AMTEOS, APTMOS, APBA *CS*: TEOS *Support*: CPE/GR	Ethanol/-	Lamotrigine	DFT: B3LYP/6-31G+; PCM—solvation model	Gaussian	15 prepolymerization complexes with individual, binary, ternary and quaternary mixture of monomers in solvent; Interaction energy calculated	[77]
	PAPhTMOS, AMTEOS, APTMOS, APBA *Support*: CPE/MWCNTs	Ethanol/-	Tinidazole	DFT: B3LYP/6-31G+; PCM—solvation model	Gaussian	15 prepolymerization complexes with individual, binary, ternary and quaternary mixture of monomers in solvent; Interaction energy calculated	[78]
	ATEOS, TEOS, TMOS *CS*: TEOS, TMOS	Mixture: ethanol + water/-	Tetracycline, ETC, EATC, ATC, OTC	PM3—conformational search of molecules, geometry optimization; HF 3-21G, SV(P), SVP—geometry optimization; MMFF94—conformational search of complexes; B3LYP/3-21G—checking that comparable structures were obtained; BSSE correction	Spartan, Gaussian	Prepolymerization complexes as systems with template:monomer ratio 1:1; Interaction energy calculated	[79]
	APTES, AEAPTMS/APTMOS, AEAPTES *CS*: TEOS *Carrier*: Fe_3_O_4_@SiO_2_	Methanol/-	Tetracycline	DFT: B3LYP 6-311G(d,p)—geometry optimization, electron densities; B3LYP-D2, M06-2X, wB97XD 6-311++G (3df,3pd)—single point calculations, UV-Vis spectra; TD-DFT—UV-Vis spectra; GIAO; BLYP/aug-cc-pVDZ—^1^H-NMR chemical shifts; AIM topology analysis; SMD—solvation model	Gaussian	Prepolymerization complexes as systems with template:monomer ratio from 1:1 to 1:3; Interaction energy calculated	[80]
Reagent ratio screening— choice the optimal ratio between reagents	APTES *CS*: TEOS *Carrier*: silica-gel microparticles	Mixture: DMSO + water/-	Valine	Polak Ribiene algorithm, AM1	Hyperchem	Prepolymerization complexes as systems with different template:monomer ratio; Interaction energy calculated	[81]
	APTES *CS*: TEOS *Carrier*: Fe_3_O_4_ capped with OA and CdTe QDs	Water/-	Ciprofloxacin	DFT: LC-WPBE/6-31G(d,p)—geometry optimization	Gaussian	Prepolymerization complexes as systems with template:monomer ratio 1:2, 1:3, 1:6; Interaction energy calculated	[82]
	APTES *CS*: TEOS *Carrier*: silicon-coated CdTe QDs	Ethanol/-	BPA	MD: COMPASS FF	Materials Studio	20 prepolymerization complexes as systems with various number of BPA, APTES, TEOS molecules; RDF calculated	[68]
	APTES *CS*: TEOS *Carrier*: silicon-coated CdTe QDs	Ethanol/-	SMX	MD: COMPASS FF	Materials Studio	10 prepolymerization complexes as systems with various number of SMX, APTES, TEOS molecules; RDF calculated	[83]
	APTES *CS*: TEOS *Carrier*: silicon-coated CdTe QDs	Ethanol/-	4NP	MD: COMPASS FF	Materials Studio	7 prepolymerization complexes as systems with various number of 4NP, APTES, TEOS molecules; RDF calculated	[84]
Explanation of the interaction way between the template and the monomer	Mixture: TEOS + PhTEOS/THS *Support*: GCE	2-ethoxy ethanol, ethanol/-	Dopamine, tyramine, dopa, epinephrine, norepinephrine	HF 3-21G	Gaussian	Prepolymerization complexes as systems with template:monomer ratio 1:1, 1:2, template:disiloxane dimer 1:1 in gas phase; Interaction energy calculated	[69]
	APTES *CS*: TEOS *Carrier*: Fe_3_O_4_@SiO_2_, Si-CD	Water/-	4NP	DFT: B3LYP/6-311G(d,p) BSSE correction	Gaussian	Prepolymerization complex as system with template:monomer ratio 1:1; Interaction energy calculated	[70]
	APTES *CS*: TEOS *Carrier and fluorescence source*: Eu/Tb-MOF	Mixture: ethanol + water/-	PFOA	MD: OPLS-AA FF (describe PFOA, APTES), SPC/E FF describe water)	LAMMPS	Prepolymerization complexes with 10 PFOA, 60 APTES, 120 TEOS, 3000 water molecules RDF calculated	[85]
	AO-DHI^+^, PETMOS, UPTMOS	Methanol/-	Naproxen	HF/6-31 G*—geometry optimization; CHelpG at the B3LYP/6-311+G(2d,2p) level—charges computing; TD-DFT—UV-Vis spectra; B3LYP, CAM-B3LYP, LC-wPBE with basis sets: 6-31++G(d,p), 6-311++G(2d,2p), cc-pVTZ—UV-Vis spectra; MD: OPLS-AA FF	Gaussian, Gromacs, VMD, Packmol	3 prepolymerization complexes with 10 template, 90 monomer, and 2471 methanol molecules; RDF, coordination number, UV-Vis spectra calculated	[86]
	APTES *CS*: TEOS	Mixture: ethanol + water/ethanol, water	1-naphthyl phosphate	DFT: M06-2x/6-31G(d,p)—geometry optimization; M06-2x/6-311++G(d,p)—free energy of the complex; SCRF, SMD—solvation model	Gaussian	Prepolymerization complex as system with template:monomer ratio 1:2; Free energy calculated	[71]
Explanation of the prepolymerization process and cavity recognition	2-mercaptonicoic acid *CS*: TEOS	-/methanol, acetonitrile, ethanol, dimethyl sulphone, water, chloroform, dichloromethane, toluene	H_3_AsO_3_	DFT: B3LYP/genecp (6-31+G(d,p) for C, H, O, N, S; Def2-TZVP for As)—geometry optimization; M06-2x/Def2TZVP—energy calculation; NCI analysis, RDG—optimal binding ratio and binding nature; AIM topology analysis; MD: UFF; PCM—solvation model; BSSE correction	Gaussian, Multiwfn, VMD, Materials Studio	Prepolymerization complexes as systems with template:monomer ratio from 1:1 to 1:5, various solvent molecules number; Cavity models with various number of CS molecules Interaction energy, RDF, selectivity coefficient calculated	[72]
Explanation of the atomistic basis of the gel matrix-template interaction	Si_3_O_3_(OH)_6_—TMOS trimer, Si_3_O_3_(OH)_5_C_3_H_6_NHC_6_H_5_—PAPhTMOS trimer	Mixtures: methanol + water/-	β-damascenone	HF/6-31G(d)—geometry optimization; CHelpG at the MP2/aug-cc-pVTZ(-f)—charges computing; MD: OPLS-AA FF	GAMESS, Gromacs, VMD	Pre-gelation models with various number of trimers, solvent molecules; RDF, coordination number calculated	[73]
	Si_3_O_3_(OH)_6_—TMOS trimer, Si_3_O_3_(OH)_5_C_3_H_6_NHC_6_H_5_—PAPhTMOS trimer *Porosity tunning agent:* PEG	Mixtures: methanol + water/-	β-damascenone	HF/6-31G(d)—geometry optimization; CHelpG at the MP2/aug-cc-pVTZ(-f)—charges computing; MD: OPLS-AA FF	GAMESS, Gromacs, VMD	Pre-gelation models with various number of trimers, solvent molecules in the presence or absence of PEG; RDF, coordination number calculated	[74]
	Si_3_O_3_(OH)_6_—TMOS trimer, [Si_3_O_3_(OH)_5_C_3_H_6_]_2_C_3_H_5_N_2_^+^—DHI^+^	Mixtures: methanol + water/-	Naproxen as anion	HF/6-31G*—geometry optimization; CHelpG at the B3LYP/6-311++G(2d,2p) level—charges computing; MD: OPLS-AA FF	GAMESS, Gromacs, VMD	Pre-gelation models with various number of solvent molecules, and trimers’ molecules in various ionic forms; RDF, coordination number calculated	[75]

Abbreviations: 4NP—4-nitrophenol, AA—acrylamide, AEAPTES—3-(2-aminoethyl)-3-aminopropyltriethoxysilane, AEAPTMS—3-(2-aminoethyl)-3-aminopropyltrimethoxysilane, AIM—atoms-in-molecules, AM1—Austin model 1, AMBER—assisted model building and energy refinement, AMTEOS—anilinomethyltriethoxysilane, AO—iodide, APBA—aminophenyl boronic acid, APTES—3-aminopropyltriethoxysilane, ATC—anhydrotetracycline, ATEOS—allyltriethoxysilane, APTMOS—3-aminopropyltrimethoxysilane, B3LYP—Becke three-parameter exchange-correlation functional, BPA—bisphenol A, BSSE—basis set superposition error, C_6_F_5_TEOS—pentafluorophenyl triethoxysilane, CNETMOS—cyanoethyltrimethoxysilane, COOTMOS—(acetoxymethyl)trimethoxysilane, COSMO—conductor like screening model, CPE—carbon paste electrode, CS—cross-linker C_5_TEOS—n-pentyltriethoxysilane, C_8_TEOS—octyltriethoxysilane, C_2_TMOS—ethyltrimethoxysilane, C_4_TMOS—n-butyltrimethoxysilane, C_8_TMOS—octyltrimethoxysilane, DFT—density functional theory, DHI^+^—1-(triethoxysilylpropyl)-3-(trimethoxysilylpropyl)-4,5-dihydroimidazolium dual cyclic cationic trimer, DMSO—dimethylsulfoxide, EATC—4-epianhydrotetracycline, ETC—4-epitetracycline, Fe_3_O_4_@SiO_2_—silica coated magnetite nanoparticles, FF—force field, GAMESS—General Atomic and Molecular Electronic Structure System, GCE—glassy carbon electrode, GIAO—gauge including atomic orbitals, GR—graphene, GTA—glutaraldehyde, HF—Hartrree–Fock, LC-WPBE—long-range corrected omega Perdew–Burke–Ernzerho, MA—methacrylamide, MAA—methacrylic acid, MD—molecular dynamics, MM—molecular mechanics, MMFF94—Merck molecular force field; MOF—metal-organic framework, MWCNTs—multi-walled carbon nanotubes, NCI—non-covalent interaction, NH_2_PhTMOS—(p aminophenyl) trimethoxysilane, NH_2_TMOS—aminotrimethoxysilane, NIPAM—N-isopropylacrylamide, OA—oleic acid, OPLS-AA—optimized potentials for liquid simulations in all atom version, OTC—oxytetracycline, PAPhTMOS—(3-propylaminophenyl)-trimethoxysilane, PCM—polarizable continuum mode, PEG—polyethylene glycol, PETMOS—4-(2-(trimethoxysilyl)ethyl)pyridine, PFOA—perfluorooctanoic acid, PhTEOS—phenyltriethoxysilane; PhTMOS—phenyltrimethoxysilane, PM3 (semi-empirical)—parametrized model 3, QD—quantum dot, R—results, RDF—radial distribution function, RDG—reduced density gradient, SCFMI—self-consistent field for molecular interactions, SCRF—self-consistent reaction field, SHTMOS—mercaptometiltrimethoxysilane, Si-CD—silane-functionalized carbon dots, SMD—solvent model density, SMX—sulfamethoxazole, SPC/E—extended simple point charge, SVP—split valence polarized, SV(P)—split valence with polarization functions on heavy atoms; TD—time dependent, TEOS—tetraethoxysilane, THF—tetrahydrofurane, THS—tetrahydroxysilane; TMCS—trimethychlorosilane, TMOS—tetramethoxysilane, UFF—universal force field, UPTMOS—ureidopropyltrimethoxysilane.

## Data Availability

No new data were created or analyzed in this study.
